# Effect of Preoperative Administration of Dexamethasone vs. Methylprednisolone in Surgical Extraction of Impacted Lower Third Molars: Randomized Controlled Clinical Trial

**DOI:** 10.3390/jcm13164614

**Published:** 2024-08-07

**Authors:** Unai Fernández-Martín, María Jesús Lisbona-González, Marta Vallecillo-Rivas, Manuel Mallo-Magariños, Francisco Javier Herrera-Briones

**Affiliations:** 1 Department of Oral Surgery and Implant Dentistry, School of Dentistry, Colegio Máximo de Cartuja s/n, University of Granada, 18071 Granada, Spain; unaifmfl@gmail.com (U.F.-M.); mvallecillo@correo.ugr.es (M.V.-R.); manu.mallo.96@gmail.com (M.M.-M.); fjherrerabriones@ugr.es (F.J.H.-B.); 2 Faculty of Dentistry, Colegio Máximo de Cartuja s/n, University of Granada, 18071 Granada, Spain

**Keywords:** dexamethasone, methylprednisolone, third molar, trismus, inflammation, postoperative pain

## Abstract

**Background/Objectives:** Glucocorticoids are drugs that are increasingly used in oral surgery to reduce trismus, inflammation, and postoperative pain, three frequent complications after the surgical extraction of impacted lower third molars. The aim of this study was to compare the effect of 8 mg dexamethasone versus 40 mg methylprednisolone in the prevention of postoperative complications after third molar surgery. **Methods**: A randomized double-blind clinical trial was conducted following CONSORT guidelines. In detail, 84 patients were included in the study, who randomly received a single preoperative submucosal dose of dexamethasone (8 mg) or methylprednisolone (40 mg). The variables analyzed, as primary outcomes, were trismus, inflammation, and postoperative pain. The measurements were performed at baseline (0 h), 3 h, 7 h, 24 h, 48 h, and 7 th day using a Visual Analog Scale (VAS), Verbal Rating Scale (VRS), and the Gabka–Matsumara method. **Results**: Dexamethasone reduced trismus, inflammation, and postoperative pain significantly better than methylprednisolone. **Conclusions**: Preoperative submucosal administration of 8 mg dexamethasone is effective and safe in reducing the severity of postoperative complications following surgical extraction of impacted lower third molars.

## 1. Introduction

Surgical extraction of impacted lower third molars is one of the most common procedures in oral surgery [1]. As an invasive intervention, an inflammatory response of variable degree appears, which depends on factors such as the patient’s individual physiological response, tissue trauma, and the degree of bone manipulation [2]. This inflammatory response is necessary for healing of the surgical area. However, when it intensifies, it can lead to postoperative complications, including inflammation (INF), trismus (TR), and postoperative pain (PP). This clinical condition has a negative impact on the quality of life of patients, interfering with daily activities such as eating and speaking, as well as making oral hygiene techniques difficult [3,4,5,6]. For this reason, the use of drugs is essential to reduce postoperative morbidity.

Nonsteroidal anti-inflammatory drugs (NSAIDs) have traditionally been used as the drugs of choice for the control of INF and PP due to their short onset of action and a prolonged analgesic duration [7,8]. Nevertheless, several clinical trials have shown that the administration of a single preoperative dose of glucocorticoids is more effective in preventing INF and PP than a single administration of NSAIDs [9,10,11].

Prostaglandins and bradykinins are the inflammatory mediators produced at the tissue injury site. The mechanism of action of corticosteroids is based on reducing the inflammatory phase by inhibiting the phospholipase A2 pathway [12]. Corticosteroids decrease the production of prostaglandins, leukotrienes, and substances related to thromboxane A2 in addition to limiting the release of lysozyme and the vasodilatory action of bradykinin [12,13]. All of this clinically represents a reduction in edema. Many studies have demonstrated the positive effect of corticosteroids to manage pain, trismus, and facial edema [13,14,15]. Trismus, following third molar surgery, has been attributed to pain, inflammation, and muscle stiffness [14]. Corticosteroids, thanks to their anti-inflammatory effect on the tissues surrounding a surgical area, have been shown to improve trismus [14,15]. Reductions in edema and trismus are associated with a decrease in inflammatory substances; however, corticosteroids do not inhibit neurotransmitters responsible for the genesis of pain, so the pain persists, although to a lesser extent [14,16].

Many routes of administration of these drugs have been described. For third molar surgery, the two most widely used have been oral and submucosal [13]. Among all routes, the oral route is the most convenient and, perhaps, the most acceptable to the patient [13]. Corticosteroids, orally administered, have been shown to undergo rapid and almost complete absorption, so their efficacy and the time it takes to reach the therapeutic plasma level are questionable. On the other hand, due to their proximity to the surgical site, corticosteroids administered submucosally could eventually act on the eicosanoids, preventing subsequent inflammatory processes [17]. Despite this, there is no clear consensus in the literature on the best route of administration [13,18].

Dexamethasone (DM) and methylprednisolone (MP) are the two most widely used corticosteroids in oral surgery due to their predominantly glucocorticoid effect and their minimal effect on sodium retention and mineralocorticoid action [19,20,21]. Both drugs can be administered by different routes, but the submucosal route has been shown to have advantages over other routes of administration: it is a less invasive technique, avoids an additional injection site, minimizes the systemic effects of the drug, and does not cause pain as it is administered over a previously anesthetized area. Hence, it has been postulated as a safe and easy route of administration that can be routinely performed in oral surgery [19,22,23,24,25].

Despite the clinically proven efficacy and the minimal, or non-existent, adverse effects with the short-term use of glucocorticoids, there is still no consensus and a clinical protocol regulating the use of these drugs in oral surgery.

With this background, the aim of this study was to compare postoperative pain, inflammation, and trismus after impacted lower third molar surgery between submucosal administration of a single preoperative dose of dexamethasone (8 mg) and methylprednisolone (40 mg). The null hypothesis was as follows: after lower third molar extraction, there are no differences, in terms of INF, TR, and PP, between patients receiving a single preoperative submucosal dose of dexamethasone (8 mg) and those receiving methylprednisolone (40 mg).

## 2. Materials and Methods

### 2.1. Design and Characteristics of the Study

#### 2.1.1. Type of Study

This double-blind randomized clinical trial was conducted at the Masters of Oral Surgery and Implant Dentistry at the University of Granada between February 2023 and June 2023.

This study was performed in accordance with the Consolidated Statement of Standards for Reporting Trials (CONSORT) [26] and complies with the Ethical Principles for Medical Research (Declaration of Helsinki, 1964), Law 41/2022 on Data Protection and Patient Rights and Law 14/2007 on Biomedical Research. In addition, this study has been approved by the Human Research Ethics Committee of the University of Granada (3180/CEIH/2023) and it is registered in clinicalTrials.gov (ID: NCT05752305).

#### 2.1.2. Study Population

All patients with an indication for surgical extraction of an impacted lower third molar and who attended the Masters of Oral Surgery and Implant Dentistry at the University of Granada (Spain) were considered for inclusion.

#### 2.1.3. Inclusion and Exclusion Criteria

Inclusion criteria were: ≥18 years old, voluntary acceptance to participate in the study and having signed the informed consent, patients with an indication for surgical extraction of one fully or partially impacted lower third molar of intermediate-high difficulty (5 points or more) using the Pederson scale and need for osteotomy [27], body mass index of 18–30 kg/m^2^, >30 min of surgery, and ASA I–II according to the American Society of Anesthesiologists. The exclusion criteria were ASA III, IV, or V status, body mass index of <18 or >30 kg/m^2^, malnutrition, not having signed the informed consent to participate in the study, and patients treated with glucocorticoids in the last 3 months or with NSAIDs in the last 7 days. Patients allergic to glucocorticoids or with contraindication for glucocorticoids administration due to their medical condition were immediately excluded. Written informed consent was obtained from each participant after a detailed explanation of the study, and participants were free to withdraw from the study at any time in accordance with the Declaration of Helsinki.

#### 2.1.4. Sample Size and Sequence

The sample size and power analysis were determined by performing a power test using G*Power software v 3.1.9.6 (Heinrich-Heine-Universität Düsseldorf, Düsseldorf, Germany). For a confidence interval of 95%, a statistical power of 90%, and a significance of *α* = 0.05, a total sample size of 84 patients was calculated. The 84 patients were divided into two groups: group 1, consisting of 42 patients who received 8 mg DM, and group 2, consisting of other 42 patients who received 40 mg MP.

#### 2.1.5. Randomization and Blinding

Patients were included in the study following a computer-generated randomization sequence. A random code (A or B) was assigned to each patient, according to the study group. The allocation was stored in sealed and numbered envelopes. To ensure blinding, both the opening of the envelopes and the preparation of the medication were performed by a person not involved in the study. Hence, the surgeon, the patient, and the operator who performed the measurements were blinded.

#### 2.1.6. Clinical Protocol

**Figure 1:**
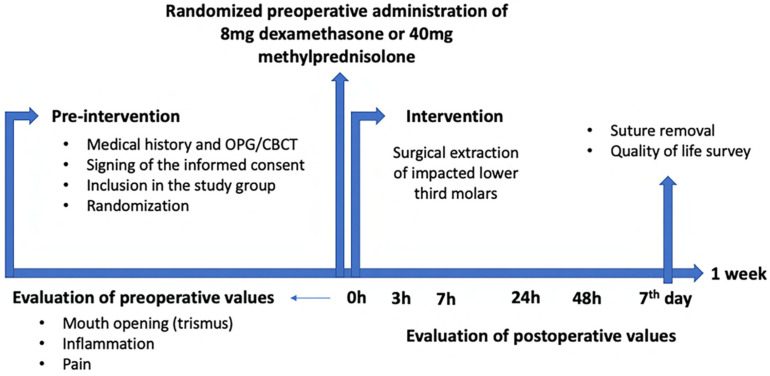
Study design: clinical protocol.

The same surgical protocol was always followed. Prior to the day of surgery, all patients underwent a detailed medical history and an intraoral and a radiological examination (orthopantomography). An additional CBCT (Cone Beam Computed Tomography) (Planmeca ProMax^®^ 3D Classic, Planmeca, Helsinki, Finland) was performed in very specific cases when the surgeon had a clinical question and considered that conventional imaging could not resolve it, such as the intimate relationship between the inferior alveolar canal and the third molar, superimpositions, and the position and impaction level of third molar. Then, patients were informed of the objective of the study and all those who voluntarily wished to participate signed an informed consent and were subsequently randomly included in one of the two groups using a randomization table. On the day of surgery, an initial assessment of the preoperative values (maximum mouth opening, inflammation, and pain) was performed by a qualified operator (M.V.-R.) in this field. Also, patients were carefully instructed on how to take these measurements in order to record the results on the data collection tables provided to them during the week following surgery. One researcher (M.J.L.-G.) was always responsible for collecting the tables. One week after surgery, sutures were removed and tables were collected, with the results recorded by the patients. The 7th day measurements were performed by the same qualified operator. Finally, patients completed a quality-of-life survey following the 14-item oral health impact profile (OHIP-14) and rating the responses from 0 to 4 (0 = never; 1 = hardly ever; 2 = occasionally; 3 = fairly often; and 4 = very often/every day) (Figure 1).

#### 2.1.7. Surgical Procedure and Medication

All the surgical interventions were performed by the same surgeon (U.F.-M.). Patients participating in the study randomly received a single preoperative submucosal dose of 8 mg DM or 40 mg MP, after blocking the inferior alveolar nerve with troncular anesthesia, 4% articaine with 1:100,000 epinephrine (Ultracain; Normon, Madrid, Spain). The surgery was performed following a standardized technique in all cases. First, depending on the difficulty, a linear or bayonet incision was made with a number 15 scalpel blade. A full-thickness flap was performed in order to completely expose the third molar and the surrounding bone. Then, ostectomy was carried out with a handpiece with a number 3 bur and odontosection with a turbine and an odontosection bur (Ökodent 016) with abundant irrigation. The third molar was luxated and removed with S3 and S4 luxators. Proper bone regularization and irrigation of the socket with saline solution were performed. Finally, flap closure was performed by repositioning the flap and suturing it with simple stitches using 3/0 silk suture (Normon, Madrid, Spain). After the surgery, all patients received postoperative instructions and were prescribed with 400 mg of ibuprofen every 8 h for the first 48 h to control INF and PP. As antibiotic therapy, 750 mg of amoxicillin every 8 h for 7 days was also prescribed or 300 mg of clindamycin for penicillin-allergic patients.

### 2.2. Outcomes Analyzed

#### 2.2.1. Primary Outcomes

The main variables analyzed in this study were TR, INF, and PP. TR was determined by measuring the interincisal distance with the patient in maximum mouth opening (millimeters). To obtain more precise results, TR measurements were performed three times at each measurement time. The mean mouth opening was then calculated. On the other hand, both INF and PP were measured using VRS and VAS. In the VRS method, four numerical values were assigned to different degrees of INF and PP, 0 being absence of inflammation/pain, 1 mild inflammation/pain, 2 moderate inflammation/pain, 3 severe inflammation/pain. For the VAS method, patients were asked to assign a numerical value to the degree of INF and PP represented on a scale, where 0 was absence of inflammation/pain and 100 was extreme inflammation/pain [28]. In addition, the Gabka and Matsumara method was also performed in the assessment of INF, measuring the distance between 5 facial anatomical points: tragus–chin, tragus–labial commissure and mandibular angle–angle of the eye distance (millimeters) [29]. All these variables were measured prior to surgery (baseline) and during the postoperative period, at 3 h, 7 h, 24 h, 48 h, and 7th day. All the results were recorded in a data collection table given to the patients.

#### 2.2.2. Secondary Outcomes

The age and sex of patients, the type of incision made (linear or bayonet), the need or not for odontosection, the number of suture stitches, and the occurrence of postoperative complication or adverse events were also considered. Patients were also asked to record the total number of NSAID tablets consumed during the postoperative period, as well as if they had to consume additional drugs to control the symptoms. On the other hand, patients completed the OHIP-14 to assess the alterations suffered in their quality of life because of having undergone the surgery.

### 2.3. Statistical Analysis

Statistical analysis was carried out by a Statistician using SPSS v 29.0 software (SPSS Inc., Chicago, IL, USA). Centralization and dispersion ratios (arithmetic mean, standard deviation, and median) were performed for descriptive analysis of the variables under study by drugs and other factors (incision and odontosection). The normality of the variables was checked using the Shapiro-Wilk test. Qualitative variables were described using relative frequencies (%) and contingency tables. An intra-drug comparative analysis was performed using the Wilcoxon test, and the Mann–Whitney test was applied for comparisons between medications. Fisher’s test was used for the study of the association between drugs and the use of rescue medication. The significance level, *α*, was set at 0.05 for all tests.

## 3. Results

**Figure 2:**
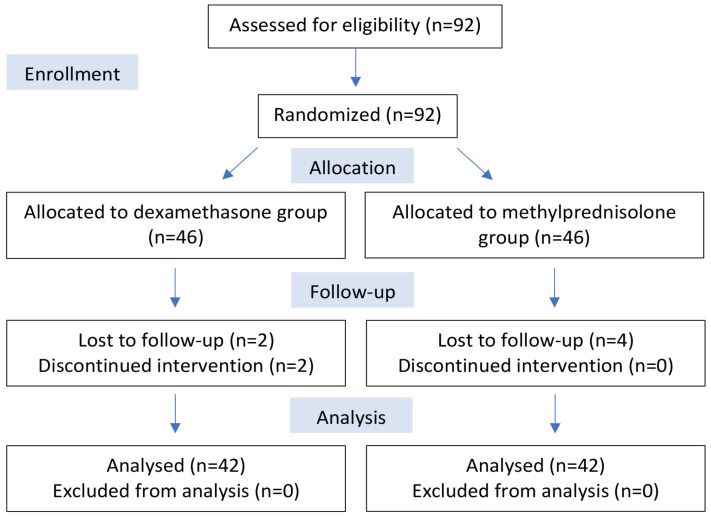
CONSORT diagram showing the flow of participants in this study.

In total, 84 participants completed the study, with a total of 8 missing patients who did not complete the data collection tables (Figure 2).

During this study, no adverse events to the drugs used or postoperative complications (alveolitis) related to the surgery were detected. The descriptive variables of this study are shown in Table 1.

### 3.1. Primary Outcomes: Trismus, Inflammation, and Pain

**Figure 3:**
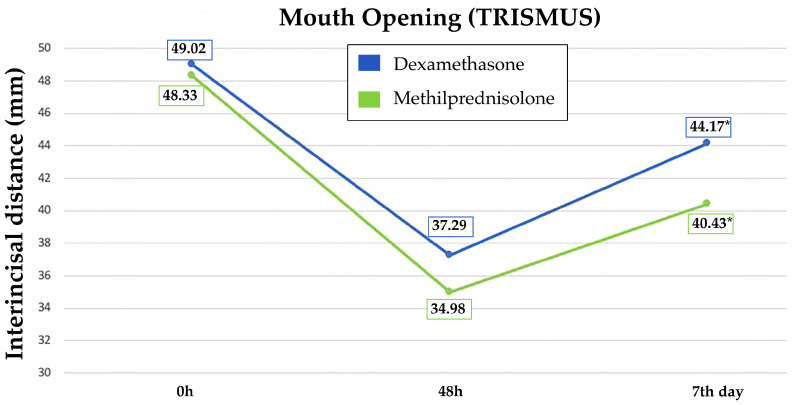
Evolution of trismus (mean values) in DM and MP groups at 0 h, 48 h and 7th day. Significant differences are shown with asterisks (*).

The mean preoperative mouth opening of the patients was 49.02 mm in the DM group and 48.33 mm in the MP group, with a statistically significant change in TR on the 7th postoperative day in favor of the DM group (44.17 mm vs. 40.43 mm, *p* = 0.018) (Table 2) (Figure 3).

**Figure 4:**
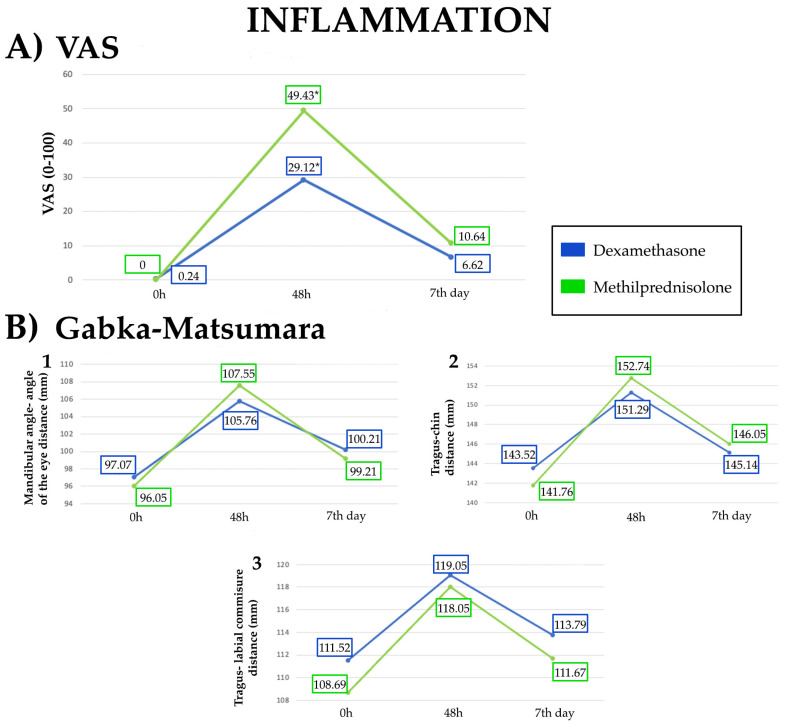
Evolution of inflammation in DM and MP groups at 0 h, 48 h and 7th day. (**A**) VAS score for inflammation (ranging from 0 to 100) in both groups. (**B**) Gabka and Matsumara method for inflammation (mean values expressed in millimeters) in both groups: (**B1**). Mandibular angle–angle of the eye distance; (**B2**). Tragus–chin distance; (**B3**). Tragus–labial commissure distance. Significant differences are shown with asterisks (*).

The highest VAS score for INF occurred at 48 h. At this time, patients in the DM group reported a statistically significant lower score than MP group (29.12 points vs. 49.43 points, *p* = 0.001). Postoperative measurement of INF, with the Gabka and Matsumara method, were lower for the DM group, although the differences were not statistically significant (Table 3) (Figure 4).

**Figure 5:**
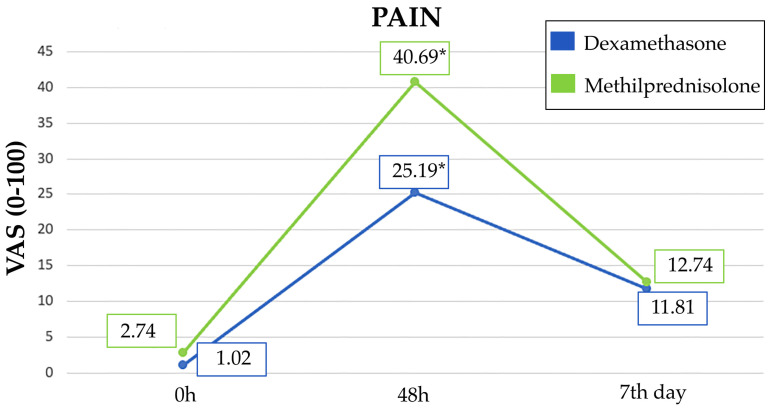
Evolution of pain (VAS score ranging from 0 to 100) in DM and MP groups at 0 h, 48 h, and 7th day. Significant differences are shown with asterisks (*).

The highest VAS score for PP occurred at 48 h. At this time, patients in the DM group reported a statistically significant lower score than MP group (25.19 points vs. 40.69 points, *p* = 0.006). However, there were no statistically significant differences between the two study groups in terms of postoperative medication consumption (Table 4) (Figure 5).

### 3.2. Secondary Outcomes

When measuring secondary outcomes, such as type of incision and the need or not for odontosection, with the primary variables (TR, INFL, and PP), statistically significant differences were detected between the two study groups. Patients in the DM group, who received a bayonet incision, showed lower TR at 7th day (44.54 mm vs. 39.64 mm, *p* = 0.009), a lower VAS score for INF at 48 h (31.03 points vs. 57.14 points, *p* = 0.00) and at 7th day (6.37 points vs. 13.11 points, *p* = 0.019), and a lower VAS score for PP at 48 h (25.37 points vs. 41.61 points, *p* = 0.009). When a linear incision was made, the tragus–chin distance was also lower in the DM group, both at 48 h (142 mm vs. 154.29 mm, *p* = 0.010) and at 7th day (139.71 mm vs. 148.50 mm, *p* = 0.016). In those cases where odontosection was carried out, patients in the DM group suffered lower TR at postoperative 7th day (43.89 mm vs. 38.71 mm, *p* = 0.023) and had lower VAS scores for INF (32 points vs. 63.90 points, *p* = 0.002) and for PP (17.79 points vs. 44.52 points, *p* = 0.004) at 48 h (Table 5, Table 6, Table 7 and Table 8).

Finally, neither drug showed statistically significant superiority over the other in reducing postoperative morbidity, measured by the quality-of-life survey. The mean score obtained in the DM group was 5.64 ± 2.76 and 7.22 ± 3.07 in the MP group, out of a maximum of 14 points.

## 4. Discussion

Our results showed that a submucosal administration of a single preoperative dose of 8 mg DM controlled better TR, INF, and PP than 40 mg MP. Therefore, the null hypothesis was rejected.

Although several studies support the benefit of glucocorticoids administration during impacted lower third molar surgery, as a preventive measure, there is still no consensus on the most effective drug type, dosage, and optimal route of administration [14,30,31].

Potential oral adverse effects of corticosteroids administration include delayed wound healing and increased risk of infection, which are usually only seen with prolonged use of these drugs [14,32,33,34]. However, in the current study, and in agreement with the scientific literature available, no adverse events to the medication used were detected after administration of a single dose of the drug before surgery. The timing of drug administration may also play a pivotal role in the final effect of the drug. The activation of the first mediators of the inflammatory response occurs immediately after the surgical incision and, depending on the route of administration, the biological effect of the drug begins within 1–2 h. For this reason, postoperative administration may be too late to fully benefit from its anti-inflammatory action [35,36,37].

For all these reasons, in our study we compared the effect of DM versus MP, the two most used corticosteroids in oral surgery, at equivalent doses (8 mg and 40 mg, respectively) administered as a single preoperative dose [4,14]. Furthermore, we did not find any study that analyzes the relationship between postoperative complications related to the type of incision and the performance of odontosection during surgery, variables that we have analyzed.

Chugh et al. 2018 observed a similar effect of DM and MP on TR control [19]. However, in our study, DM was more effective in controlling TR throughout the postoperative period, especially on 7th day, which is in accordance with the study carried out by Srivastava et al. 2021 [35]. Furthermore, in other clinical trials in which the same corticosteroids were administered orally, instead of injected, and with the same doses, greater mouth opening was also shown when DM was used [4,5].The maximum peak of INF detected in the study was at 48 h, with statistically significant results in favor of the use of DM. DM demonstrated a greater control of INF, especially on the second postoperative day: a result that agrees with other authors [4,5,19,35].The greater effectiveness of DM compared to MP in controlling TR and INF is probably because DM is a long half-life (36–72 h) corticosteroid with a greater anti-inflammatory potency (relative potency of 30) compared to MP, which has an intermediate pharmacological effect (12–36 h) and a relative potency of 5 [22,34].

In the present investigation, postoperative edema and inflammation were measured by various methods, using VRS and VAS, but also with the Gabka and Matsumara method. Scales, such as VRS and VAS, may have the limitation of being influenced by subjectivity, so completing them with a quantitative measurement in millimeters allows us not only to overcome this limitation but also to increase the certainty of the measurements. The Gabka and Matsumara method used in this investigation for inflammation assessment (tape measurements by marking of certain facial points) has been stated in the literature as a valid, non-invasive, easy, and inexpensive method [4,35,38,39]. Currently, these forms of evaluation have been replaced by three-dimensional (3D) optical scanning techniques [38,40,41]. It seems that 3D techniques demonstrated greater homogeneity than the manual method when analyzing facial distortions caused by the same swelling simulation; however, there are many authors who report a lack of standardization in the 3D measurement points [41,42].

PP is a complication that can negatively affect patients’ quality of life. However, postoperative quality of life was minimally affected using both drugs, with no statistically significant differences between them. Unlike other authors [4,5,19,35] who did not find statistically significant differences between DM and MP in the control of PP, in our study DM showed a greater reduction in PP at 48 h, although, as in the aforementioned studies, no significant differences were found in the consumption of postoperative medication between the two groups.

On the other hand, we observed that patients who underwent bayonet incision and/or odontosection, and received DM, showed lower TR, INF, and PP. This is probably due, in addition to the pharmacological properties of DM, to the fact that performing a bayonet incision considerably improves access to and vision of the surgical area, and the vertical incision could act as a drainage route for inflammatory exudate.

The main limitation when conducting this study was establishing a clinical protocol that standardized the dose, the route of glucocorticoids administration, and data collection. The assessment of these variables is relatively subjective, and bias and heterogeneity between studies is described in meta-analyses [14,23]. Other limitations of this study may be derived from its methodology. The present work was performed with a relatively small sample, although it was adequate to produce statistically significant results. Furthermore, the absence of a placebo group could be considered an additional limitation, since a placebo effect on the effect of the postoperative variables measured cannot be ruled out. Finally, measuring variables such as those studied in this investigation (trismus, inflammation) is always a challenge, although this potential weakness was addressed by using standardized categorical instruments such as VRS and VAS and considering multiple outcome methods.

After reviewing the literature and based on the results obtained in our study, we believe that there is sufficient scientific evidence to recommend the administration of glucocorticoids (8 mg DM) in impacted lower third molar surgery. Corticosteroids have been shown to be an effective and safe medication in preventing TR, INF, and PP, although further studies are required to confirm these findings and to determine the optimal dose of DM for this purpose.

## 5. Conclusions

In conclusion, DM was demonstrated to be better for avoiding TR during the postoperative period, and to be more effective in controlling INF and PP after impacted third molar extraction. In addition, when bayonet incision and/or odontosection were performed, the use of DM significantly reduced TR, INF, and PP. Based on the results obtained, this study shows that a single preoperative submucosal dose of DM, used as a short-term glucocorticoid, appears to be a promising strategy in oral surgery.

## Figures and Tables

**Table 1:** Summary of the descriptive variables of the study.

Variables	Dexamethasone (*n* = 42)	Methylprednisolone (*n* = 42)	Total (*n* = 84)
Age (mean ± SD)	23.74 ± 6.09	23.60 ± 3.77	23.67 ± 5.04
Sex (*n* (%))	Men: 20 (47.6%) Women: 22 (52.4%)	Men: 19 (45.2%) Women: 23 (54.8%)	Men: 39 (46.4%) Women: 45 (53.6%)
Number of suture stitches (mean ± SD)	4.10 ± 1.01	3.98 ± 1.22	4.04 ± 1.11
Incision (*n* (%))	Linear: 7 (16.70%) Bayonet: 35 (83.3%)	Linear: 14 (33.30%) Bayonet: 28 (66.7%)	Linear: 21 (25%) Bayonet: 63 (75%)
Odontosection (*n* (%))	No: 23 (54.8%) Yes: 19 (45.2%)	No: 21 (50%) Yes: 21 (50%)	No: 44 (52.4%) Yes: 40 (47.6%)

SD = standard deviation.

**Table 2:** Preoperative and postoperative values of mouth opening (trismus).

		Dexamethasone (*n* = 42)			Methylprednisolone (*n* = 42)		
Mouth Opening (TRISMUS)		Preoperative (0 h)	Maximum 48 h	Maximum 7th Day	Preoperative (0 h)	Maximum 48 h	Maximum 7th Day
Interincisal distance (mm)	Mean ± SD	49.02 ± 6.92	37.29 ± 9.62	44.17 ± 7.66	48.33 ± 6.75	34.98 ± 9.10	40.43 ± 7.52
	Median	50.00	38.50	43.00	48.00	33.50	39.00
	*p*-value	0.575	0.280	0.018 *	0.575	0.280	0.018 *

SD = standard deviation. * Statistical significance.

**Table 3:** Preoperative and postoperative values of inflammation.

		Dexamethasone (*n* = 42)			Methylprednisolone (*n* = 42)		
INFLAMMATION		Preoperative (0 h)	48 h	7th Day	Preoperative (0 h)	48 h	7th Day
**VAS** (0–100)	Mean ± SD	0.24 ± 1.54	29.12 ± 25.72	6.62 ± 11.22	0.00 ± 0.00	49.43 ± 27.99	10.64 ± 12.05
	Median	0.00	23.00	0.00	0.00	50.00	5.50
	*p*-value	0.317	0.001 *	0.108	0.317	0.001 *	0.108
**Gabka and Matsumara method**		Preoperative (0 h)	48 h	7th day	Preoperative (0 h)	48 h	7th day
Tragus–chin distance (mm)	Mean ± SD	143.52 ± 12.52	151.29 ± 12.59	145.14 ± 10.29	141.76 ± 11.37	152.74 ± 10.10	146.05 ± 9.13
	Median	140.50	149.50	143.00	142.50	151.00	145.00
	*p*-value	0.982	0.387	0.247	0.982	0.387	0.247
Tragus–labial commissure distance (mm)	Mean ± SD	111.52 ± 8.07	119.05 ± 12.27	113.79 ± 7.90	108.69 ± 7.11	118.05 ± 9.19	111.67 ± 7.52
	Median	110.00	117.00	113.00	110.00	117.00	110.50
	*p*-value	0.157	0.879	0.240	0.157	0.879	0.240
Mandibular angle–angle of the eye distance (mm)	Mean ± SD	97.07 ± 8.49	105.76 ± 10.27	100.21 ± 9.14	96.05 ± 7.98	107.55 ± 12.01	99.21 ± 7.51
	Median	97.00	106.00	100.50	95.00	107.00	99.00
	*p*-value	0.493	0.597	0.496	0.493	0.597	0.496

SD = standard deviation. * Statistical Significance.

**Table 4:** Preoperative and postoperative values of pain.

		Dexamethasone (*n* = 42)				Methylprednisolone (*n* = 42)			
PAIN		Preoperative (0 h)		48 h	7th Day	Preoperative (0 h)		48 h	7th Day
**VAS** (0–100)	Mean + SD	1.02 ± 4.84		25.19 ± 24.91	11.81 ± 17.05	2.74 ± 7.98		40.69 ± 26.36	12.74 ± 13.73
	Median	0.00		20.00	5.00	0.00		30.00	10.00
	*p*-value	0.659		0.006 *	0.381	0.659		0.006 *	0.381
**Postoperative medication**		Patients (%)		Mean (tablets) ± SD	Median	Patients (%)		Mean (tablets) ± SD	Median
		Yes	No			Yes	No		
Ibuprofen		42 (100%)	0 (0%)	13.40 ± 5.47	15.00	42(100%)	0(0%)	14.64 ± 5.23	15.00
Acetaminophen		14 (33.3%)	28 (66.7%)	1.43 ± 2.84	0.00	9 (21.4%)	33 (78.6%)	0.76 ± 1.66	0.00
*p*-value		0.328							

SD = standard deviation. * Statistical significance.

**Table 5:** Results of trismus related to the type of incision and odontosection.

		Dexamethasone (*n* = 42)		Methylprednisolone (*n* = 42)	
Incision		Bayonet	Linear	Bayonet	Linear
Preoperative (0 h) maximum mouth opening	Patients (*n*)	35	7	28	14
	Mean ± SD	49.49 ± 7.13	46.71 ± 5.59	49.43 ± 6.09	46.14 ± 7.67
	*p*-value (intra-drug)	0.370		0.436	
	*p*-value (inter-drug)	0.857	0.799	0.857	0.799
Maximum mouth opening at 48 h	Mean ± SD	37.26 ± 9.85	37.43 ± 9.03	34.04 ± 10.08	36.88 ± 6.68
	*p*-value (intra-drug)	0.817		0.189	
	*p*-value (inter-drug)	0.212	0.856	0.212	0.856
Maximum mouth opening at 7th day	Mean ± SD	44.54 ± 8.04	42.49 ± 5.41	39.64 ± 8.04	42.00 ± 6.32
	*p*-value (intra-drug)	0.388		0.218	
	*p*-value (inter-drug)	0.009 *	0.971	0.009 *	0.971
**Odontosection**		No	Yes	No	Yes
Preoperative (0 h) maximum mouth opening	Patients (*n*)	23	19	21	21
	Mean ± SD	49.52 ± 8.03	48.42 ± 5.43	48.00 ± 8.80	48.67 ± 3.97
	*p*-value (intra-drug)	0.604		0.630	
	*p*-value (inter-drug)	0.638	0.630	0.638	0.630
Maximum mouth opening at 48 h	Mean ± SD	39.04 ± 10.39	35.16 ± 8.37	37.67 ± 9.85	32.29 ± 7.59
	*p*-value (intra-drug)	0.287		0.072	
	*p*-value (inter-drug)	0.588	0.270	0.588	0.270
Maximum mouth opening at 7th day	Mean ± SD	44.39 ± 9.14	43.89 ± 5.60	42.14 ± 7.86	38.71 ± 6.52
	*p*-value (intra-drug)	0.543		0.151	
	*p*-value (inter-drug)	0.269	0.023 *	0.269	0.023 *

SD = standard deviation. * Statistical significance.

**Table 6:** Results of inflammation (VAS) related to the type of incision and odontosection.

		Dexamethasone (*n* = 42)		Methylprednisolone (*n* = 42)	
Incision		Bayonet	Linear	Bayonet	Linear
Preoperative (0 h) VAS inflammation	Patients (*n*)	35	7	28	14
	Mean ± SD	0.29 ± 1.69	0.00 ± 0.00	0.00 ± 0.00	0.00 ± 0.00
	*p*-value (intra-drug)	0.921		1.000	
	*p*-value (inter-drug)	0.371	1.000	0.371	1.000
VAS inflammation at 48 h	Mean ± SD	31.03 ± 25.14	19.57 ± 28.48	57.14 ± 22.50	34.00 ± 32.17
	*p*-value (intra-drug)	0.181		0.026 *	
	*p*-value (inter-drug)	0.00 *	0.322	0.00 *	0.322
VAS inflammation at 7th day	Mean ± SD	6.37 ± 11.25	7.86 ± 11.85	13.11 ± 12.32	5.71 ± 10.16
	*p*-value (intra-drug)	0.895		0.076	
	*p*-value (inter-drug)	0.019 *	0.799	0.019 *	0.799
**Odontosection**		No	Yes	No	Yes
Preoperative (0 h) VAS inflammation	Patients (*n*)	23	19	21	21
	Mean ± SD	0.43 ± 2.09	0.00 ± 0.00	0.00 ± 0.00	0.00 ± 0.00
	*p*-value (intra-drug)	0.363		1.000	
	*p*-value (inter-drug)	0.339	1.000	0.339	1.000
VAS inflammation at 48 h	Mean ± SD	26.74 ± 20.92	32.00 ± 30.92	34.95 ± 22.65	63.90 ± 25.56
	*p*-value (intra-drug)	0.741		0.001 *	
	*p*-value (inter-drug)	0.206	0.002 *	0.206	0.002 *
VAS inflammation at 7th day	Mean ± SD	5.70 ± 8.80	7.74 ± 13.77	9.14 ± 11.46	12.14 ± 12.71
	*p*-value (intra-drug)	0.801		0.442	
	*p*-value (inter-drug)	0.493	0.169	0.493	0.169

SD = standard deviation. * Statistical significance.

**Table 7:** Results of inflammation (Gabka and Matsumara method) related to the type of incision and odontosection.

		Dexamethasone (*n* = 42)		Methylprednisolone (*n* = 42)	
Incision		Bayonet	Linear	Bayonet	Linear
Preoperative (0 h) tragus–chin distance (mm)	Patients (*n*)	35	7	28	14
	Mean ± SD	145.46 ± 12.20	133.86 ± 9.86	140.43 ± 11.04	144.43 ± 11.97
	*p*-value (intra-drug)	0.034 *		0.163	
	*p*-value (inter-drug)	0.250	0.016 *	0.250	0.016 *
Tragus–chin distance (mm) at 48 h	Mean ± SD	153.14 ± 12.74	142.00 ± 6.56	151.96 ± 10.54	154.29 ± 9.34
	*p*-value (intra-drug)	0.028 *		0.518	
	*p*-value (inter-drug)	0.776	0.010*	0.776	0.010 *
Tragus–chin distance (mm) at 7th day	Mean ± SD	146.23 ± 10.70	139.71 ± 5.74	144.82 ± 9.51	148.50 ± 8.08
	*p*-value (intra-drug)	0.205		0.189	
	*p*-value (inter-drug)	0.901	0.016 *	0.901	0.016 *
Preoperative (0 h) tragus–labial commissure distance (mm)	Mean ± SD	112.60 ± 8.13	106.14 ± 5.43	107.86 ± 7.48	110.36 ± 6.21
	*p*-value (intra-drug)	0.045 *		0.228	
	*p*-value (inter-drug)	0.022 *	0.172	0.022 *	0.172
Tragus–labial commissure distance (mm) at 48 h	Mean ± SD	120.06 ± 12.75	114.00 ± 8.54	117.64 ± 10.68	118.86 ± 5.36
	*p*-value (intra-drug)	0.205		0.802	
	*p*-value (inter-drug)	0.673	0.128	0.673	0.128
Tragus–labial commissure distance (mm) at 7th day	Mean ± SD	114.80 ± 8.05	108.71 ± 4.79	110.89 ± 8.22	113.21 ± 5.85
	*p*-value (intra-drug)	0.053		0.208	
	*p*-value (inter-drug)	0.061	0.224	0.061	0.224
Preoperative (0 h) mandibular angle–angle of the eye distance (mm)	Mean ± SD	97.54 ± 8.96	94.71 ± 5.44	95.68 ± 7.39	96.79 ± 9.29
	*p*-value (intra-drug)	0.353		1.000	
	*p*-value (inter-drug)	0.421	0.535	0.421	0.535
Mandibular angle–angle of the eye distance (mm) at 48 h	Mean ± SD	106.46 ± 10.23	102.29 ± 10.53	108.61 ± 12.90	105.43 ± 10.12
	*p*-value (intra-drug)	0.353		0.420	
	*p*-value (inter-drug)	0.570	0.689	0.570	0.689
Mandibular angle–angle of the eye distance (mm) at 7th day	Mean ± SD	100.66 ± 9.36	98.00 ± 8.25	98.82 ± 6.87	100.00 ± 8.89
	*p*-value (intra-drug)	0.446		0.989	
	*p*-value (inter-drug)	0.353	0.585	0.353	0.585
**Odontosection**		No	Yes	No	Yes
Preoperative (0 h) tragus–chin distance (mm)	Patients (*n*)	23	19	21	21
	Mean ± SD	143.43 ± 10.13	143.63 ± 15.22	142.00 ± 10.46	141.52 ± 12.47
	*p*-value (intra-drug)	0.849		0.753	
	*p*-value (inter-drug)	1.000	0.979	1.000	0.979
Tragus–chin distance (mm) at 48 h	Mean ± SD	149.04 ± 9.10	154.00 ± 15.67	150.90 ± 9.18	154.57 ± 10.85
	*p*-value (intra-drug)	0.462		0.213	
	*p*-value (inter-drug)	0.564	0.688	0.564	0.688
Tragus–chin distance (mm) at 7th day	Mean ± SD	144.26 ± 7.79	146.21 ± 12.83	145.95 ± 7.76	146.14 ± 10.52
	*p*-value (intra-drug)	0.558		0.632	
	*p*-value (inter-drug)	0.409	0.668	0.409	0.668
Preoperative (0 h) tragus–labial commissure distance (mm)	Mean ± SD	110.26 ± 6.84	113.05 ± 9.31	108.38 ± 6.72	109.00 ± 7.63
	*p*-value (intra-drug)	0.447		0.860	
	*p*-value (inter-drug)	0.458	0.236	0.458	0.236
Tragus–labial commissure distance (mm) at 48 h	Mean ± SD	115.35 ± 6.92	123.53 ± 15.68	118.10 ± 8.95	118.00 ± 9.65
	*p*-value (intra-drug)	0.094		0.880	
	*p*-value (inter-drug)	0.323	0.376	0.323	0.376
Tragus–labial commissure distance (mm)at 7th day	Mean ± SD	113.04 ± 7.43	114.68 ± 8.54	110.80 ± 6.50	112.52 ± 8.49
	*p*-value (intra-drug)	0.780		0.520	
	*p*-value (inter-drug)	0.334	0.486	0.334	0.486
Preoperative (0 h) mandibular angle–angle of the eye distance (mm)	Mean ± SD	97.43 ± 8.12	96.63 ± 9.12	96.29 ± 7.63	95.81 ± 8.49
	*p*-value (intra-drug)	0.639		0.480	
	*p*-value (inter-drug)	0.715	0.573	0.715	0.573
Mandibular angle–angle of the eye distance (mm) at 48 h	Mean ± SD	105.30 ± 10.40	106.32 ± 10.37	106.95 ± 11.96	108.14 ± 12.33
	*p*-value (intra-drug)	0.790		0.734	
	*p*-value (inter-drug)	0.621	0.789	0.621	0.789
Mandibular angle–angle of the eye distance (mm) at 7th day	Mean ± SD	100.52 ± 9.28	99.84 ± 9.22	98.00 ± 6.71	100.43 ± 8.23
	*p*-value (intra-drug)	0.790		0.261	
	*p*-value (inter-drug)	0.340	0.915	0.340	0.915

SD = standard deviation. * Statistical significance.

**Table 8:** Results of postoperative pain related to the type of incision and odontosection.

		Dexamethasone (*n* = 42)		Methylprednisolone (*n* = 42)	
Incision		Bayonet	Linear	Bayonet	Linear
Preoperative (0 h) pain (VAS)	Patients (*n*)	35	7	28	14
	Mean ± SD	1.23 ± 5.29	0.00 ± 0.00	3.04 ± 8.96	2.14 ± 5.79
	*p*-value (intra-drug)	0.644		0.927	
	*p*-value (inter-drug)	0.990	0.636	0.990	0.636
Pain (VAS) at 48 h	Mean ± SD	25.37 ± 24.62	24.29 ± 28.35	41.61 ± 25.72	38.86 ± 28.51
	*p*-value (intra-drug)	0.817		0.664	
	*p*-value (inter-drug)	0.009 *	0.287	0.009 *	0.287
Pain (VAS) at 7th day	Mean ± SD	11.89 ± 17.56	11.43 ± 15.47	13.21 ± 14.05	11.79 ± 13.53
	*p*-value (intra-drug)	0.921		0.702	
	*p*-value (inter-drug)	0.323	0.913	0.323	0.913
**Odontosection**		No	Yes	No	Yes
Preoperative (0 h) pain (VAS)	Patients (*n*)	23	19	21	21
	Mean ± SD	0.48 ± 2.09	1.68 ± 6.87	0.48 ± 2.18	5.00 ± 10.72
	*p*-value (intra-drug)	0.804		0.134	
	*p*-value (inter-drug)	0.628	0.630	0.628	0.630
Pain (VAS) at 48 h	Mean ± SD	31.30 ± 27.52	17.79 ± 19.54	36.86 ± 21.55	44.52 ± 30.49
	*p*-value (intra-drug)	0.088		0.449	
	*p*-value (inter-drug)	0.345	0.004 *	0.345	0.004 *
Pain (VAS) at 7th day	Mean ± SD	10.04 ± 15.42	13.95 ± 19.05	12.62 ± 10.37	12.86 ± 16.70
	*p*-value (intra-drug)	0.968		0.396	
	*p*-value (inter-drug)	0.142	0.915	0.142	0.915

SD = standard deviation. * Statistical significance.

## Data Availability

The original contributions presented in this study are included in the article, and further inquiries can be directed to the corresponding author.

## Abbreviations

DM	dexamethasone
INF	inflammation
MP	methylprednisolone
NSAIDs	nonsteroidal anti-inflammatory drugs
PP	postoperative pain
TR	postoperative pain
VAS	Visual Analog Scale
VRS	Verbal Rating Scale
